# Auditing the diagnosis of cancer in primary care: the experience in Scotland

**DOI:** 10.1038/sj.bjc.6605397

**Published:** 2009-12-03

**Authors:** P Baughan, B O'Neill, E Fletcher

**Affiliations:** 1 Forth Valley Lead Cancer Team, Falkirk and District Royal Infirmary, Trust HQ, Westburn Avenue, Falkirk FK1 5SU, UK; 2 Chair of Scottish Primary Care Cancer Group, Springwell House, Edinburgh, UK; 3 Health Intelligence Unit, NHS Lothian, Edinburgh, UK

**Keywords:** primary care, delays in diagnosis, referral pathway

## Abstract

**Introduction:**

This paper reports on an ongoing primary care audit of cancer referrals undertaken in Scotland in 2006–2007 and 2007–2008.

**Methods:**

General practitioners (GPs) in Scotland were asked to review all new cancer diagnoses within their practice during the preceding year.

**Results:**

4181 patients were identified in year 1 and 12 294 in year 2. The pathway taken for patients to present to, and be referred from, their GP has been analysed for 7430 of the 12 294 patients identified within year 2 across five separate health boards. The time from first symptoms to presentation to a GP varied between tumour types, being the longest (median 30 days) for head and neck cancers and the shortest (median 2 days) for bladder cancer. In all, 25% of patients within the following tumour groups waited longer than 2 months to present to their GP following first symptoms: prostate, colorectal, melanoma and head and neck cancers. Once patients had presented to their GP, those with prostate and lung cancer were referred later (median time 11 days) than those with breast cancer (median time 2 days). The priority with which GPs referred patients varied considerably between tumour groups (breast cancer 77.5% ‘urgent’ compared with prostate cancer 44.7% ‘urgent’). In one health board the proportion of cancer patients being referred urgently increased from 46% to 58% between the first and second audit.

**Conclusion:**

Our data show that there are very different patterns of presentation and referral for patients with cancer, with some tumour groups being more likely to be associated with a delayed diagnosis than others.

Early presentation, prompt investigation and timely access to definitive treatment unquestionably improves the experience of patients, although there is some doubt about when and whether delays affect outcomes with many cancers (Richards *et al*, 1999; Jensen *et al*, 2007; Neal *et al*, 2007; Chen *et al*, 2008; Hamilton, 2009).

Different countries have different systems for allowing access to specialist services. In the United Kingdom, other than diagnoses made through the three screening services (breast, cervical and more recently, colorectal cancers), patients usually first present to their general practitioners (GPs). GPs have an important function in assessing which patterns of symptoms are most likely to be suggestive of cancer. If cancer is suspected, patients may be referred immediately on first presentation for specialist assessment and investigations, or they may have initial investigations and review within primary care and then be referred when the results of tests are available.

Specific guidelines have been developed in Scotland and elsewhere to support GPs in referral decisions for patients with suspected cancer (National Institute for Health and Clinical Excellence, 2005; Scottish Executive Health Department (SEHD), 2007; Scottish Government, 2009).

Targets have been introduced in an attempt to ensure that patients suspected of having cancer are seen and investigated promptly, then fast-tracked for definitive treatment to cure or palliate their cancer. In both England (Secretary of State for Health, 1997) and Scotland (SEHD, 2001), there is a 62-day target from referral to treatment for patients whose referral is marked urgent and for those referred as an emergency (including self-referral to accident and emergency departments). In England (but not in Scotland), there is also a 2-week target from urgent referral to first assessment by specialist services (Secretary of State for Health, 1997).

The aim of this study is to gain a better understanding of how quickly patients with cancer initially present to their GP, and how they are then referred to secondary care for further investigation and treatment.

## Materials and methods

On two separate occasions between 2005 and 2008, GPs in most health boards in Scotland were asked to review all new cancer diagnoses within their practice during the preceding year. It was hoped that by engaging practices in a cancer-review process, it would be possible to consolidate knowledge around the early parts of the cancer journey including typical presentation of cancers, symptom development and when and how to refer for further investigation. The first audit took place in 2006–2007 and involved reviewing all patients diagnosed with cancer in 2005–2006; the second took place in 2007–2008, relating to patients diagnosed in 2006–2007. Engagement with the study was facilitated by the enhanced service component of the General Medical Services Contract, which enabled a payment for participating practices.

Participating GPs across Scotland were asked to review the clinical notes of each new patient diagnosed with cancer and were given guidance regarding how to record the items listed in Table 1 onto a standard electronic template. They were then asked to reflect on the patient journey and to comment on ways that it could have been improved. Patients with cancer detected through the national screening programmes were excluded from the study, as were those with non-melanoma skin cancer.

Data were available from five health boards across Scotland during the first review, and from nine health boards during the second (Scotland is served by 11 mainland and three island health boards), and work is currently underway to amalgamate these data across the whole of Scotland. The results in this paper relate to the initial analysis of the second year's data, taken from five of the nine participating health boards. Comparative data from one health board relating to priority of referral over the two separate years of the audit have also been included.

## Results

In all, 4181 patients were identified with a new diagnosis of cancer during year 1 and 12 294 in year 2. This compares with a total of ∼27 000 new cases of cancer diagnosed each year in Scotland (Information Services Division, 2008). In the first year of the audit, each health board collected data in different ways, making collation difficult. Arrangements in year 2 were more systematic, allowing data to be collected on 12 294 patients with cancer. Detailed analysis has been conducted on data for 7430 of the 12 294 patients. Data on the remaining 4864 patients are not yet analysed.

**Figure 1:**
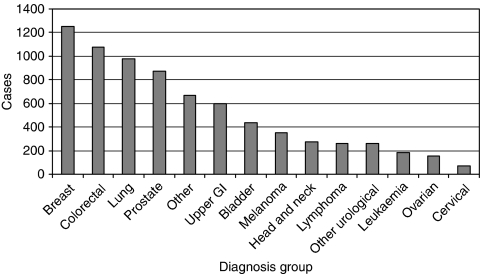
Distribution of cancers within analysis.

The 7430 cases analysed were identified by 540 GP practices from five different health boards in Scotland. The cases covered all major tumour types (Figure 1; Table 2) and reflected a similar pattern to that reported nationally (Information Services Division, 2008).

This paper focuses on the analysis of the following:

- time from patient first noticing symptoms to first presentation with a GP,
- time from first presentation to time of referral,
- priority of referral from primary to secondary care.

### Time from patient first noticing symptoms to first presentation with a GP

**Figure 2:**
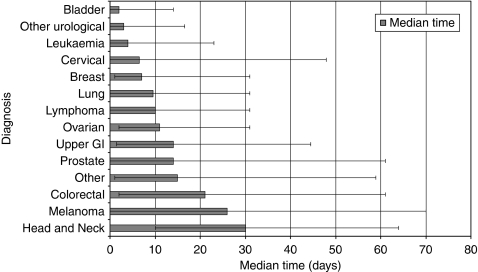
Median time from first noticing symptoms to first presentation with a GP.

The time taken for patients to present to a GP varied according to tumour site (Figure 2; Table 3). Patients with head and neck cancer took the longest to present (median time 30 days). Patients with melanoma (median time 26 days) and colorectal cancer (median time 21 days) also presented comparatively late. In all, 25% of patients with the following cancers waited longer than 1 month before first presenting: breast, lung, lymphoma, ovarian and upper gastrointestinal. For prostate, colorectal, melanoma, and head and neck cancers, the same proportion of patients, 25%, waited 2 months or more to first present to a GP.

The shortest times between first noticing a sign or symptom and first presentation to a GP were for patients with bladder cancer (median time 2 days), leukaemia (4 days), cervical cancer (6.5 days) and breast cancer (7 days).

### Time from first presentation to time of referral

**Figure 3:**
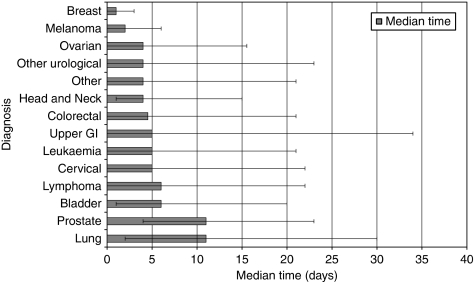
Median time from first presentation to time of referral.

The time taken for a GP to refer a patient with a suspicion of cancer also varied according to tumour group (Figure 3; Table 4). Patients with breast cancer and melanoma were referred quickly (median times 1 day and 2 days, respectively), whereas for other tumour groups (notably lung and prostate), patients spent much longer within the primary care part of the journey before being referred to secondary care (lung cancer 11 days, prostate cancer 11 days). In all, 25% of patients with lung cancer and upper gastrointestinal cancer were not referred for 1 month or more following initial presentation.

### Priority of referral from primary to secondary care

**Figure 4:**
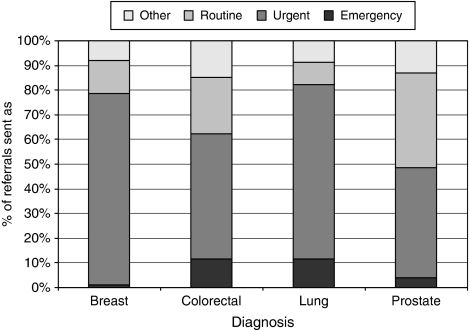
Priority of referral by tumour group for breast, colorectal, lung and prostate cancers.

One of the most important factors determining time to diagnosis was the priority with which the GP sent the referral. When the referral priority was examined for the four most common cancers (Figure 4; Table 5), a much higher proportion of patients with breast cancer (969; 77.5%) and lung cancer (694; 70.7%) were referred ‘urgently’ to secondary care compared with colorectal cancer (543; 50.6%) and prostate cancer (391; 44.7%). Patients with colorectal and lung cancer were more likely than prostate or breast cancer patients to present as an emergency admission, and of these four tumour groups, patients with prostate cancer had the highest likelihood of being referred to hospital ‘routinely’ (337; 38.6%). The category ‘other’ included referrals that were marked as ‘soon’ and referrals to private hospitals or clinics.

**Figure 5:**
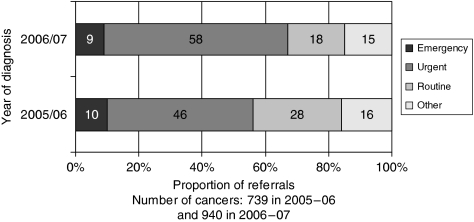
Proportion of patients referred by priority group within one health board over 2 successive years.

When referral priority data were compared within one of the five health boards over the two separate time periods (following an intensive GP education programme), the proportion of all cancers presenting to GPs that were referred ‘urgently’ increased from 340 out of 739 referrals (46%) in 2005–2006 to 545 out of 940 referrals (58%) in 2006–2007 (Figure 5). The difference in the total number of cancers diagnosed from year 1 to year 2 is accounted for by a slight increase in the number of GP practices taking part in the audit during the second year.

**Figure 6:**
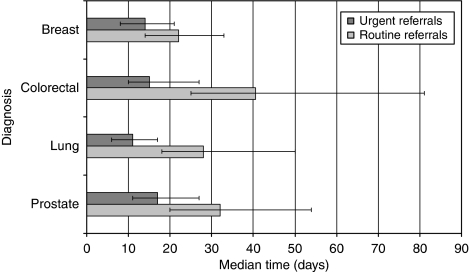
Median time from referral sent to first seen by specialist.

The extent to which the priority of referral contributed to delays in diagnosis was evident when the time to first see a hospital specialist was examined. When the four commonest tumour groups were examined, the time taken to see a specialist was considerably longer if the patient was referred routinely (Figure 6; Table 6). The median time for a patient with lung cancer to see a specialist was 11 days for an urgent referral, yet 28 days for a routine referral.

## Discussion

This study has yielded valuable information about the primary care pathway for over 16 000 patients diagnosed with cancer in Scotland over two separate periods. Detailed analysis of 7430 patients from five separate health boards has been reported in this paper. This has highlighted differences in the way that individual cancers present to, and are referred by, GPs.

Patients with head and neck cancers, melanomas and colorectal cancers waited comparatively longer before seeking help from their GP. When the inter-quartile ranges were examined, it is apparent that 25% patients with prostate cancer, colorectal cancer, melanoma and head and neck cancers took longer than 2 months to present to a GP following the first symptom or sign of cancer. Limited research has been done on what causes patients to delay presenting for advice or referral (Ramirez *et al*, 1999; Burgess *et al*, 2001), but it is clear that many patients are not aware of the common symptoms and signs that might suggest a diagnosis of cancer. Although there have been occasional public education and other campaigns to raise awareness and encourage early presentation, there is little objective measurement of their effectiveness. A recent study examined attempts to positively influence and subsequently evaluate interventions to encourage early presentation of women with breast cancer (Burgess *et al*, 2009).

GPs can influence the time from first presentation to referral. The delay in referral for both lung and prostate cancer patients can be explained by recommendations that initial assessments and investigations be completed before referral (e.g. chest X-ray in suspected lung cancer and evaluation of prostate-specific antigen in prostate cancer) (SEHD, 2007).

For some tumour groups, less than half of all newly diagnosed cancers were referred urgently. Of the four commonest tumour groups, marked differences were noted between the proportions of breast cancer patients referred urgently (969; 77.5%) compared with colorectal (543; 50.6%) and prostate cancer patients (391; 44.7%).

The importance of referring a patient with cancer ‘urgently’ is that these patients are actively ‘fast-tracked’ through the hospital diagnostic system to ensure compliance with the 62-day target from urgent referral to treatment (SEHD, 2001). As ‘routine’ and ‘soon’ referrals are not subject to these targets, they are not prioritised and invariably take much longer to start treatment following the date of referral.

With this audit, GPs were given the opportunity to comment on each individual patient's pathway to diagnosis. On reflection, many GPs indicated that they should have referred their patient more urgently than they did; however, the most common explanation from GPs was that the patient did not have the classic symptoms and signs described within the urgent cancer referral guidelines. Referral guidelines for some tumours may not always favour patients with early symptoms of cancer. One study (Neal *et al*, 2007) found that for lung cancer (a tumour with a poor prognosis), referral guidelines were prioritising those with more advanced disease. However, the same was not found for patients with colorectal, ovarian or prostate cancer.

Despite doubt about the benefit of urgent referral pathways, one encouraging finding was the change in the proportion of patients referred urgently within one health board during the two separate years of the study. This change was also noted within several different health boards and may reflect increasing awareness of guidelines on the part of referrers and the increased priority that cancer has been given in Scotland in recent years. Data are awaited from a further audit (again through the enhanced service component of the General Medical Services Contract) to examine the compliance of all urgent suspected cancer referrals with current referral guidelines.

By engaging with 540 different GP practices across five health boards, there is a risk of variable data capture depending on the thoroughness with which individual GPs reviewed their clinical notes. The development of clear guidance for data collection and the rigorous checking of all data submitted will have helped to reduce this variability. However, by engaging with GPs across Scotland in the collection of these data, it has been possible to facilitate education around the typical presentation of cancer. Comments written by GPs undertaking this audit provide a wealth of information. Individual practices were frequently very open about their shortcomings and appeared to provide perceptive analysis of the diagnostic journey. Significant event analysis has become an embedded part of reflective learning by GPs and forms a component of the annual appraisal system. Data from this audit facilitated significant event analyses within many of the GP practices taking part.

Although doubt has been cast on the benefit of cancer waiting time targets, whether 2-week waits or 62-day targets (Jones *et al*, 2001), and some have shown a perverse adverse effect on breast cancer referrals (Potter *et al*, 2007), public opinion and published evidence supports the benefit of prompt recognition, prompt referral and early effective treatment for patients with cancer. Primary care health professionals have an important function in early diagnosis.

## Figures and Tables

**Table 1:** Components of cancer diagnosis review

Patient diagnosis
Date patient first noticed symptoms
Date patient first reported symptoms to primary care
Date of decision to refer
Date referral sent
Priority given to referral (e.g. emergency, urgent, routine)
Use of any specific cancer referral *pro forma*
Method of sending referral (e.g. electronic, secure fax, post)
Date patient first seen by specialist
Date patient told the diagnosis
Date GP informed of diagnosis
Reflective comments on patient pathway through primary care

Abbreviation: GP=general practitioner.

**Table 2:** Distribution of cancers within analysis

**Diagnosis group**	**Number of referrals**
Bladder	439
Breast	1250
Cervical	69
Colorectal	1074
Head and neck	273
Leukaemia	181
Lung	981
Lymphoma	260
Melanoma	353
Other	667
Other urological	258
Ovarian	152
Prostate	874
Upper GI	599

Abbreviation: GI=gastrointestinal.

**Table 3:** Median time from first noticing symptoms to first presentation with a GP

**Diagnosis group**	**Median time (days)**	**Inter-quartile range (days)**
Bladder	2.0	14.0
Breast	7.0	30.0
Cervical	6.5	48.0
Colorectal	21.0	59.0
Head and neck	30.0	54.0
Leukaemia	4.0	23.0
Lung	9.5	31.0
Lymphoma	10.0	31.0
Melanoma	26.0	70.0
Other	15	58.0
Other urological	3.0	16.5
Ovarian	11.0	29.0
Prostate	14.0	61.0
Upper GI	14.0	43.0

Abbreviation: GI=gastrointestinal.

**Table 4:** Median time from first presentation to time of referral

**Diagnosis group**	**Median time (days)**	**Inter-quartile range (days)**
Bladder	6.0	19.0
Breast	1.0	3.0
Cervical	5.0	22.0
Colorectal	4.5	21.0
Head and neck	4.0	14.0
Leukaemia	5.0	21.0
Lung	11.0	28.0
Lymphoma	6.0	22.0
Melanoma	2.0	6.0
Other	4.0	21.0
Other urological	4.0	23.0
Ovarian	4.0	15.5
Prostate	11.0	19.0
Upper GI	5.0	34.0

Abbreviation: GI=gastrointestinal.

**Table 5:** Priority of referral by tumour group for breast, colorectal, lung and prostate cancers

	**% of referrals sent as priority**			
**Diagnosis**	**Emergency**	**Urgent**	**Routine**	**Other**
Breast	1.0	77.5	13.5	8.0
Colorectal	11.7	50.6	23.0	14.8
Lung	11.5	70.7	9.1	8.7
Prostate	3.8	44.7	38.6	12.9

Due to the effects of rounding, row totals may not equal 100% exactly.

**Table 6:** How referral priority influenced time to see specialist

	**Median time to see specialist (days)**	
**Diagnosis**	**Routine**	**Urgent**
Breast	22.0	14.0
Colorectal	40.5	15.0
Lung	28.0	11.0
Prostate	32.0	17.0
